# When a Histologic Diagnosis Becomes Factitious: A Case of Munchausen Syndrome

**DOI:** 10.7759/cureus.28876

**Published:** 2022-09-07

**Authors:** Mafalda Teixeira, Pedro Santos, Ana Bastos Furtado, José Delgado Alves

**Affiliations:** 1 Department of Internal Medicine IV, Hospital Professor Doutor Fernando Fonseca, Amadora, PRT; 2 Department of Internal Medicine IV, Systemic Immune-Mediated Diseases Unit (UDIMS), Hospital Professor Doutor Fernando Fonseca, Amadora, PRT

**Keywords:** c-peptide, exogenous hyperinsulinism, hypoglycemia, adult nesidioblastosis, factitious disorder

## Abstract

Recurrent episodes of hypoglycemia are uncommon in non-diabetic patients. The workup investigation must confirm hypoglycemia and distinguish between endogenous versus exogenous hyperinsulinism. Simultaneous measurements of plasma glucose, insulin, C-peptide, and a screen for oral hypoglycemic agents should be performed. According to the results, further imaging studies may be necessary. A 43-year-old woman suffering from recurrent hypoglycemia presented to the emergency room (ER) with a hypoglycemic coma. She has had multiple episodes of documented hypoglycemia for the last 13 years. The case was initially investigated, and laboratory studies revealed endogenous hyperinsulinism. Screening for sulfonylureas, anti-insulin and anti-insulin receptor antibodies were negative. Body imaging and positron emission tomography (PET) with ^68^Ga-DOTANOC did not show evidence of an insulinoma. The patient was submitted to a pancreatectomy, which revealed nesidioblastosis in the histologic examination. Since then, the patient became hyperglycemic but the insulin doses were progressively reduced until new episodes of hypoglycemia recurred and the insulin was stopped. Again, inappropriately high levels of insulin were found at the time of hypoglycemic episodes. Computed tomography (CT) and PET scans did not find evidence of an insulinoma. A C-peptide was later found to be negative and insulin ampoules were found in her possession, making a diagnosis of a factitious disorder. Although rare, factious disorders are frequently overlooked and challenging to diagnose. Since they are very resource and time-consuming, self-inflicted illnesses should always be considered and ruled out beforehand.

## Introduction

Recurrent episodes of hypoglycemia are uncommon in non-diabetic patients [1,2]. When in the presence of a documented hypoglycemia without diabetes mellitus, it is important to review the history, physical findings, and all available laboratory data seeking clues to specific disorders [1]. When the cause of the hypoglycemic disorder is not evident, it is crucial to measure plasma glucose, insulin, C-peptide, and beta-hydroxybutyrate concentrations and screen for oral hypoglycemic agents (ideally all available sulfonylureas) during an episode of spontaneous hypoglycemia [1,3]. This investigation will distinguish hypoglycemia caused by endogenous (or exogenous) insulin from that caused by other mechanisms [3] (see Appendix 1, Table 4).

In a seemingly healthy individual, the differential diagnosis narrows to two general categories: endogenous hyperinsulinism (see Appendix 2, Table 5) and accidental/surreptitious hypoglycemia.

In adults, hyperinsulinemic hypoglycemia (HH) accounts for 0.5-5% of cases of hypoglycemia and can be due either to β-cell tumors (insulinomas) or β-cell hyperplasia [3].

Insulinoma, the most common cause of hypoglycemia related to endogenous hyperinsulinism, occurs in one to four people per million of the general population [4]. The noninsulinoma pancreatogenous hypoglycemia syndrome (NIPHS) is a rare cause of persistent hyperinsulinemic hypoglycemia in adults and its frequency is much less than that of insulinoma. The incidence of nesidioblastosis in adults is unknown but is generally thought to be very low [5]. Nesidioblastosis is the histological equivalent of NIPHS and refers to an increase in the size and number of pancreatic beta cell islets with focal or diffuse hypertrophy and hyperfunction [5].

## Case presentation

A 43-year-old woman with a previous history of recurrent hypoglycemia presented to the emergency room after being found unconscious at home due to severe hypoglycemia. She had a known medical history of autoimmune keratitis with a prolonged history of systemic corticosteroid therapy for which she underwent bilateral cornea transplantation in 2007. 

Initial diagnosis

The history of the present illness began in July 2008. She was 30-year-old at the time, unemployed and living with her parents in a suburban town near Lisbon. She was brought to the emergency room with a loss of consciousness due to severe hypoglycemia. In the beginning, hypoglycemia was admitted as secondary to iatrogenic adrenal insufficiency due to prolonged corticosteroid therapy, and she was started on hydrocortisone. Medical records from that time are scarce and we could not find an accurate description of the pattern of hypoglycemia (besides some references to them being more severe during fasting and happening throughout the day) or the diagnostic work-up that was performed. The patient ended up being discharged and continued to be monitored in an ambulatory setting. There are limited records of what happened in the following years, but the patient was re-admitted to our hospital in 2013 due to severe and persistent hypoglycemia.

Hypoglycemia pattern

During that hospital stay, it was possible to document recurrent severe hypoglycemia, which had an erratic pattern, happening throughout the day, mostly during fasting. The patient required continuous glucose infusion, especially during the night when, sometimes and due to more severe hypoglycemias, she ended up needing rescue glucagon administrations, to which she had a good response.

Diagnostic workup

Laboratory studies revealed endogenous hyperinsulinism-inappropriately high insulin levels during a hypoglycemic episode and insulin to C-peptide ratio inferior to 1 (Table 1). Surreptitious administration of sulfonylureas was excluded with the available sulfonylurea assay that tests for glibenclamide, tolbutamide, and chlorpropamide. Anti-insulin and anti-insulin receptor antibodies were also negative. Insulin-like growth factor 1 (IGF-1) dosing was within the normal range. Free fatty acids, beta-hydroxybutyrate, and acetoacetate were inappropriately low for the glucose plasma concentration, which was consistent with hyperinsulinism.

**Table 1:** Insulin, C-peptide, insulin/C-peptide ratios, and IGF-1 levels measurements during hypoglycemic episodes throughout the years. Beta-hydroxybutyrate levels were not consistently measured and are not presented here. IGF-1: insulin-like growth factor 1.

	2008		2013			2014		2020		2021	
Glucose (mg/dL)	28	52	32	25	49	48	65	42	50	27	36
Insulin (mUI/L)	46.4	6.1	54.2	31.9	61.4	9.4	4.5	<0.4	<0.4	11.4	6.1
Insulin (pmol/L)	322.2	42.6	376.6	221.5	426.4	65.5	31.5	<2.8	<2.8	79.2	42.2
C-peptide (ug/L)	12.8	2.6	6.0	4.8	5.3	0.9	0.8	<0.1	<0.1	<0.1	-
C-peptide (pmol/L)	4250.2	866.7	1992.3	1603.8	1753.2	295.5	252.4	33.2	33.2	33.2	-
Insulin/C-peptide ratio	0.076	0.049	0.189	0.138	0.243	0.222	0.125	-	-	>2.3	-
IGF-1 (ng/mL)	247	168	217	-	-	104	124	-	-	200.4	-

Regarding the differential diagnosis of endogenous hyperinsulinism, full body computed tomography (CT) scans were performed without evidence of a neuroendocrine tumor (NET), such as an insulinoma. She was then submitted to endoscopic pancreatic ultrasonography that excluded a pancreatic tumor's presence. She also underwent nuclear imaging targeting somatostatin receptors-^68^Ga-DOTANOC positron emission tomography (PET)-which did not reveal any abnormal metabolic uptake suggestive of a hormone-producing neuroendocrine tumor (NET).

In the pursue of NIPHS, she was further submitted to selective pancreatic arteriography followed by intra-arterial pancreatic calcium stimulation testing (Table 2). In this invasive procedure, arteries supplying the pancreas are cannulated with a subsequent injection of intra-arterial calcium gluconate, a pancreatic secretagogue that stimulates insulin release. A greater than twofold increase in the hepatic venous insulin levels over baseline can help to regionalize an insulinoma, especially if imaging is negative. In this case, an increase in the hepatic venous insulin levels was found after calcium administration in the superior mesenteric artery. These results suggest the existence of a hyperfunctioning pancreatic area that could be an insulinoma or a noninsulinoma pancreatogenous hypoglycemia syndrome (NIPHS).

**Table 2:** Results of the selective arterial calcium stimulation test. Intra-arterial calcium was injected into the three selected arteries that contribute to the blood supply of different pancreatic regions. A twofold or higher increase in insulin concentration (in contrast to no response from normal β-cells) in the supra-hepatic vein, in response to the injection of calcium gluconate is considered to indicate an insulinoma in the vascular territory of the artery studied. The area supplied by the superior mesenteric artery shows a fourfold increase in insulin levels after calcium stimulation.

Insulin levels (mUI/L)	Before	30 minutes after	60 minutes after	120 minutes after
Splenic artery	10.49	14.14	15.00	3.11
Gastroduodenal artery	12.53	17.24	20.93	17.86
Superior mesenteric artery	6.00	26.67	27.28	17.83

Treatment

Considering the possibility of an occult pancreatic insulinoma or hyperfunctioning pancreatic tissue, medical therapy was first attempted and the patient was treated with diazoxide, which was up-titrated to 700 mg per day, and octreotide, which was up-titrated to 200 mcg per day.

Due to the medical refractoriness, she was proposed for a partial pancreatectomy, with intraoperative pancreatic ultrasonography to aid the search for a pancreatic insulinoma which she accepted. A corpo-caudal pancreatectomy was performed with a negative intraoperative ultrasound screening. Histologic examination found no evidence of an insulinoma but rather an increased number of pancreatic islets of Langerhans, many with increased size and irregular contours consistent with nesidioblastosis. She was diagnosed with NIPHS.

Clinical course after diagnosis and treatment

After surgery, as expected, the patient developed diabetes and needed insulin to achieve glycemic control. However, six months later, she began to describe new spells of neuroglycopenia associated with low plasma glucose concentration and ended up discontinuing insulin administration. The lab work was, once again, consistent with endogenous hyperinsulinism (Table 3).

**Table 3:** Proposed histological criteria for the diagnosis of NIPHS in adults. NIPHS: noninsulinoma pancreatogenous hypoglycemia syndrome.

Proposed histological criteria for the diagnosis of NIPHS in adults
Exclusion of an insulinoma by macroscopic, microscopic, and immunohistochemical examination.
Multiple beta cells with an enlarged and hyperchromatic nucleus and abundant clear cytoplasm.
Islets with the normal spatial distribution of the various cell types.
No proliferative activity of endocrine cells.

Due to the severity of the recurrent hypoglycemia, she was started again on diazoxide and octreotide, the latter being changed to pasireotide due to its higher potency as a somatostatin analog and higher hyperglycemic effects. Appropriate glycemic control was achieved, and she remained stable for some time.

In 2016, three years after the first surgery, due to surgical and medical therapies refractoriness, she underwent a duodenopancreatectomy. Again, histological examination of the remaining pancreatic tissue revealed an increase in pancreatic islets of Langerhans, many with increased size and irregular contours consistent with nesidioblastosis.

She ended up developing diabetes (“again”) and pancreatic exocrine insufficiency, for which she was medicated with insulin and pancreatin.

Hypoglycemia strikes back

In 2020, four years after this last episode, hypoglycemic episodes recurred and insulin was again stopped. The lab work from that time revealed absent levels of insulin and C-peptide (Table 1). Although inconsistent with the lab findings, she underwent new abdominal CT imaging and ^68^Ga-DOTANOC PET functional imaging with no evidence of remaining pancreatic tissue or an extra-pancreatic neuroendocrine tumor. Regarding medical management, she was started on diazoxide with a marginal response but was able to return home. 

The most recent hospital admission

In 2021, after multiple episodes of severe hypoglycemia and no response to medical therapy, she was once more admitted to the hospital. She relied on glucose perfusions even though severe episodes of hypoglycemia still occurred during her hospital stay, requiring multiple glucagon boluses. 

The lab work showed inappropriately high insulin levels during hypoglycemia (Table 1). Abdominal magnetic resonance imaging (MRI) and ^18^F-DOPA PET scans did not show evidence of an insulinoma or ectopic pancreatic tissue. C-peptide levels were later found to be absent (confirmation of the results took two weeks to become available). Based on the confounding results, a factious disorder became a possibility. The patient was later found to have insulin ampoules, becoming evident that this was a case of self-induced hypoglycemia. When confronted with the facts, the patient vehemently denied self-administering insulin or any other drug. She was put under surveillance and, after 24 to 48 hours, normoglycemia was achieved and the intravenous glucose infusion stopped. Since she had no unexpected puncture marks on her skin, we assumed she administered insulin through her central venous catheter. In fact, her central line was changed about four times since her admission (approximately two weeks), always due to the early development of inflammatory signs at the insertion site. 

After the diagnosis, she began psychotherapy and was referred to psychiatry. Since her mother became the person in-charge of her glycemic control and insulin administration, hypoglycemic episodes have not been reported or recurred to date.

## Discussion

This case presented initially with a documented Whipple’s triad: inappropriately high plasma insulin and C-peptide levels, no detectable glibenclamide, tolbutamide, and chlorpropamide levels during hypoglycemia and no circulating antibodies to insulin. A diagnosis of hyperinsulinemic hypoglycemia (HH) was correctly made and the search for the most common cause of HH in adults-an insulinoma-began. Abdominal CT and endoscopic pancreatic ultrasonography, which combined have a sensitivity greater than 90% in some centers, failed to identify a pancreatic tumor, so a selective pancreatic arterial calcium stimulation test was performed and found a positive result after the cannulation of the superior mesenteric artery. Considering there was the persistence of severe and recurrent hypoglycemia despite maximal medical therapy, subtotal pancreatectomy was proposed. Intraoperative pancreatic ultrasonography was once again negative and, guided by the results of the calcium stimulation test, a corpo-caudal pancreatectomy was performed.

It is very difficult to diagnose nesidioblastosis based on clinical and imaging features. Nesidioblastosis cannot be differentiated from insulinoma both clinically and biochemically, which makes it almost impossible to diagnose before surgery [6]. The final diagnosis relies on the pathologic analysis of the pancreatic tissue [7] (Table 3). 

A diagnosis of NIPHS was made, and the patient was discharged from the hospital requiring insulin to achieve glycemic control. 

Half a year later, there was a recurrence of neuroglycopenia spells, and insulin was stopped. At that time, laboratory studies revealed endogenous hyperinsulinism. After another attempt at medical treatment, the patient ended up being submitted for a total pancreatectomy to control hypoglycemia. Again, histological examination of the pancreatic tissue was compatible with nesidioblastosis.

The treatment of adult nesidioblastosis is partial resection of the pancreas [8]. Since the first reported case of nesidioblastosis in 1975, fewer than 100 patients have been described, which means little is known regarding the efficacy of medical and surgical treatment, especially in adults. Nevertheless, it is reported that a distal pancreatectomy of 60% to 80% is usually successful in controlling hypoglycemia in most patients [9]. So far, it is impossible to predict the recurrence of hypoglycemia in adult patients with nesidioblastosis.

In our case, although rare, nesidioblastosis appeared to be refractory to partial pancreatectomy, and a multidisciplinary team decided that a total pancreatectomy was the only option to control the disease.

Four years after the second surgery, the patient was admitted once again due to the recurrence of hypoglycemia. This time, laboratory work revealed hypoglycemia with absent plasma levels of insulin and C-peptide, which was expected for someone submitted to a total pancreatectomy.

One year later, she was readmitted with hypoglycemia and the workup showed inappropriately high insulin levels during hypoglycemia. C-peptide levels were measured and confirmed in a different lab, which took about two weeks to become available. During this time, the patient required high rates of intravenous glucose infusion to maintain normoglycemia as well as several glucagon boluses when hypoglycemia was critical. Considering the severity of hypoglycemia, the patient underwent new abdominal imaging (CT, MRI, and PET with ^Ga^68-DOTANOC) that found no evidence of remaining pancreatic tissue or an extrapancreatic neuroendocrine tumor. When the C-peptide levels were available and proven to be undetectable, a diagnosis of exogenous hyperinsulinism was suspected, and insulin ampoules and needles were found among the patient’s belongings. 

After becoming obvious that this was a case of self-induced hypoglycemia, there were many aspects of the clinical course and management that we reflected upon. First, although adult nesidioblastosis is extremely rare, it is a clinical entity to consider in the differential diagnosis of hypoglycemia. Although not mandatory, patients with NIPHS typically have postprandial hypoglycemia [2]. This was never the case with our patient who, from the beginning, experienced random episodes of hypoglycemia that happened throughout the day and mainly during fasting.

Generally, there is no need for a total pancreatectomy in nesidioblastosis since most patients can achieve glycemic control after subtotal (75-90%) pancreatic resection [7]. We haven’t found other cases of nesidioblastosis so severe that they needed to undergo a total pancreatectomy to control the disease.

The etiology of nesidioblastosis in adults is unknown. Still, a clinical case reported in the past highlighted the possibility of this histological pattern being induced by prolonged use of sulfonylureas [10]. After an intensive investigation, we found prescriptions for glimepiride in the patient's name dating earlier than 2008. Furthermore, we also unraveled that she had easy access to this medication because her father was also taking glimepiride at that time since he was a diabetic. As there is no way to prove it, but considering the very unusual clinical course, we speculate that this extremely rare case of nesidioblastosis might actually be a consequence of a surreptitious administration of sulfonylureas. This specific type of sulfonylurea was not measured in our hospital assay, thus making it impossible to exclude this hypothesis. Screening for oral hypoglycemic agents was not repeated before the second surgery. 

When revisiting her social history, we realized this woman was forced into early retirement at 30 and did not have any occupation, as well as interests or hobbies. According to her mother, she spent her days at home with her parents, whom she lived with, and did not have any meaningful relationships besides her family's inner circle.

We strongly believe this is a dramatic and protracted case of malicious hypoglycemia in a young woman who went through a lot of invasive and expensive exams and ended up undergoing very extensive and radical surgery, even though she knew it was completely unnecessary.

Analyzing the patient’s behavior during this last admission, we came to the conclusion that she was always found unusually at ease considering her problems and unclear future perspectives. Besides, she underwent all investigations without any discomfort and always showed acknowledgment for our dedication and hard work. Playing the "sick role" allowed her to adopt an identity that brought support and acceptance from others. Being admitted to the hospital probably gave her a clearly defined place in a social network, something she did not have outside of the hospital.

According to the Diagnostic and Statistical Manual of Mental Disorders (DSM-5), “Factitious disorder imposed on self” is defined as: “Falsification of physical or psychological signs or symptoms, or induction of injury or disease, associated with identified deception (…) in the absence of obvious external rewards” [11]. Munchausen syndrome is one form of factitious disorder used to describe the more severe and chronic forms [11]. Considering our suspicion of this being a case of nesidioblastosis induced by sulfonylureas from the beginning, we may consider it a case of Munchausen syndrome that took about 13 years to be diagnosed.

## Conclusions

Factitious disorder imposed on self-Munchausen syndrome is a syndrome in which patients consciously induce, feign, or exaggerate physical or psychiatric symptoms for primary gain. Patients with factitious disorders can pose a significant danger to themselves by undergoing a plethora of unnecessary procedures, often over-utilizing limited healthcare resources. Although rare, factious disorders are frequently overlooked and pose a significant challenge for healthcare providers to make a diagnosis. Since they are very resource and time-consuming, self-inflicted illnesses should always be considered and ruled out beforehand.

## Appendices

Appendix 1

**Table 4:** Diagnostic criteria for patients with hyperinsulinaemic hypoglycemia. *These criteria assume the absence of intercurrent illnesses (sepsis or other critical illnesses like renal or hepatic failure).

Diagnostic criteria* for patients with hyperinsulinaemic hypoglycemia
Plasma glucose <55 mg/dL (<3.0 mmol/L) with:
Detectable serum insulin ≥18 pmol/L;
Detectable C-peptide ≥0.6 ng/mL (in endogenous hyperinsulinaemic hypoglycemia);
Suppressed or low serum ketone bodies (3-𝛃-hydroxybutyrate <2.0 mmol/L);
Suppressed or low serum concentrations of free fatty acids (<1.5 mmol/L).

Appendix 2

**Table 5:** Causes of hypoglycemia in adults.

Causes of hypoglycemia in adults
Ill or medicated individual:
Drugs (insulin or insulin secretagogues, alcohol, others)
Critical illnesses (hepatic, renal or cardiac failure, sepsis, inanition)
Hormone deficiency (cortisol)
Nonislet cell tumor (result of tumor overproduction of IGF-1 or IGF-2)
Seemingly well individual:
1. Endogenous hyperinsulinism:
Insulinoma
Functional 𝛃-cell disorders (nesidioblastosis): noninsulinoma pancreatogenous hypoglycemia syndrome (NIPHS); post-gastric bypass hypoglycemia
Insulin autoimmune hypoglycemia (antibody to insulin, antibody to insulin receptor)
Insulin secretagogue
2. Accidental, surreptitious, or malicious hypoglycemia
